# Hypotensive episodes associated with azithromycin infusion: a potentially fatal adverse drug reaction

**DOI:** 10.1002/rcr2.464

**Published:** 2019-08-05

**Authors:** Jeffrey Wong, Maitri Munsif, Robyn O'Hehir, Mark Hew, Eli Dabscheck

**Affiliations:** ^1^ Department of Respiratory Medicine The Alfred Hospital Melbourne Victoria Australia

**Keywords:** Antibiotics, azithromycin, hypotension, pneumonia

## Abstract

We report the first case of hypotensive episodes associated with intravenous (IV) azithromycin. This is a potentially fatal adverse drug reaction (ADR), with the risk of irreversible end‐organ damage, and therefore must be recognized and treated immediately. In our case, the reaction was successfully managed by immediate cessation of the azithromycin infusion. It is important for clinicians to be aware of this ADR as azithromycin is a commonly prescribed medication worldwide.

## Introduction

We report the first case of hypotensive episodes associated with intravenous (IV) azithromycin administration. It is a potentially fatal complication and must be recognized and treated immediately.

## Case Report

A 64‐year old male Caucasian ex‐smoker with a 9‐year history of severe interstitial lung disease (ILD) presented with dyspnoea. Blood tests showed elevated inflammatory markers, and chest imaging demonstrated a new bilateral infiltrate consistent with the diagnosis of severe community‐acquired pneumonia. He was prescribed daily IV azithromycin of 500 mg and IV ceftriaxone of 1 g and was admitted to the ward.

His past medical history included well‐controlled type 1 diabetes mellitus and psoriasis. He had no prior known drug allergies. On multiple previous admissions, the patient had received ceftriaxone and piperacillin/tazobactam (Tazocin) without complications.

On the medical ward, he became hypotensive with a drop of blood pressure from 100/70 to 60/30 mmHg. His oxygen saturation dropped from 97 to 60%, and he became tachypnoeic (26 breaths/min), peripherally cyanotic, and unresponsive with a Glasgow Coma Scale (GCS) score of 3. The patient spontaneously regained consciousness after 20 s without adrenaline use. His GCS returned to 15 within 5 min. After the episode, the patient reported feeling warm, flushed, sweaty, and nauseated. There was no associated laryngeal angioedema, rash, or fever during this episode. He was admitted to the intensive care unit (ICU) for further evaluation.

Serum troponins, sputum analysis, viral polymerase chain reaction (PCR), chest X‐ray, ventilation‐perfusion (V/Q) scan, and duplex ultrasound of the lower limbs were all unremarkable. The electrocardiogram (ECG) performed at the time of the episode and as a repeat ECG showed no arrhythmias; however, corrected QT interval (QTc) was slightly prolonged on both occasions (QTc = 471 and 491 ms, respectively). His blood glucose level was high (15.1 mmol/L); serum urea, electrolyte, and creatinine (UECs) showed mild acute kidney injury (Creatinine 114 μmol/L, estimated glomerular filtration rate 59 mL/min); and liver function tests (LFTs) were mildly deranged (Albumin = 31 g/L, alanine aminotransferase = 63 U/L, gamma‐glutamyl transferase = 229 U/L, alkaline phosphatase = 214 U/L). Serial serum tryptase measurements were within the normal limits on three occasions (immediate, 2 h, and 24‐h).

Over the course of 2 days in the ICU on the monitor, he experienced two further hypotensive episodes. Only after the third episode did his medical team realize that all episodes had occurred during the azithromycin infusion, with resolution immediately upon infusion cessation. Azithromycin was then discontinued, with no subsequent recurrences. Telemetry throughout ICU admission showed no evidence of any arrhythmias.

## Discussion

There have been no reported cases of immediate hypotensive episodes with IV azithromycin infusion in the literature. Azithromycin is widely prescribed for severe community‐acquired pneumonia. The most common adverse effects with azithromycin (both oral and IV) are gastrointestinal side effects such as nausea, diarrhoea, and abdominal pain 1, followed by hepatotoxicity 2, and QT prolongation leading to sudden cardiac death (SCD) 3.

The hypotensive episodes are likely an adverse drug reaction (ADR) following azithromycin infusion. This does not constitute a typical drug‐induced anaphylactic reaction as there was no angioedema, limited respiratory impairment beyond slight tachypnoea, and no mucocutaneous involvement. The reactions were self‐resolving without the use of adrenaline and did not cause an elevation in serial serum tryptase measurements. Drug‐induced anaphylaxis is usually associated with elevated serum tryptase. The reaction was not due to the ceftriaxone infusion as the patient had received ceftriaxone on multiple previous admissions without complications, and there was no clear temporal relationship as opposed to azithromycin.

According to the current literature, azithromycin use has not been shown to cause hypotensive episodes. However, three studies concluded that there is an increased risk of hypotension or shock with the co‐prescription of calcium‐channel blockers and macrolide antibiotics, specifically erythromycin and clarithromycin but not azithromycin 4, 5, 6. In our case, the patient was not on any concurrent antihypertensive medications.

A meta‐analysis on macrolides and cardiovascular risk showed that azithromycin use is 3.4 times more likely to cause SCD or ventricular tachyarrhythmias (VTA) 3. In our case, there were no abnormal findings on ECG.

A few case reports have also outlined Drug Rash with Eosinophilia and Systemic Symptoms (DRESS) syndrome in adults 7 and children 8, 9 upon azithromycin administration. However, in our case, there was no mucocutaneous involvement and no accompanying peripheral eosinophilia.

In summary, we report the first case of hypotensive episodes following an infusion of IV azithromycin. It is important to recognize that hypotension could result from the administration of a commonly prescribed antibiotic. It is a potentially life‐threatening condition that may lead to irreversible organ dysfunction and death if left untreated. The current consensus guidelines in managing an ADR is to immediately cease its administration, and that exact management was sufficient for our patient without requiring other measures such as adrenaline. We would like to emphasize the lessons learnt from our experience to recognize a potentially serious ADR. The awareness of this potential drug reaction could hopefully benefit future clinical practice worldwide.

### Disclosure Statement

Appropriate written informed consent was obtained for publication of this case report and accompanying images.
